# Amyand’s Hernia: An Uncommon Encounter of an Appendiceal Presence in an Inguinal Hernia

**DOI:** 10.7759/cureus.61348

**Published:** 2024-05-30

**Authors:** Akash Inamdar, Raju K Shinde, Krushank Nayak, Yogesh B Manek

**Affiliations:** 1 General Surgery, Jawaharlal Nehru Medical College, Datta Meghe Institute of Higher Education and Research, Wardha, IND

**Keywords:** surgical management, lichtenstein meshplasty, vermiform appendix, inguinal hernia, amyand's hernia

## Abstract

Amyand’s hernia is a rare variant of inguinal hernia characterized by the presence of the vermiform appendix within the hernia sac. It represents a unique diagnostic and management challenge for surgeons due to its low incidence and varied clinical presentations. Here, we present a case of a 45-year-old man with a one-year history of right inguinoscrotal swelling, diagnosed as a right indirect inguinal hernia. Preoperative imaging revealed the presence of omentum within the hernia sac. Intraoperatively, both the omentum and the vermiform appendix were found within the sac without evidence of inflammation. The patient underwent successful Lichtenstein meshplasty without appendicectomy. This case highlights the importance of considering Amyand’s hernia in the differential diagnosis of inguinal hernias and the significance of intraoperative findings in guiding surgical management. Further studies and case reports are needed to enhance our understanding of this rare clinical entity and optimize patient outcomes.

## Introduction

Inguinal hernias are common surgical conditions characterized by the protrusion of abdominal contents through a weakness or defect in the inguinal canal [1]. While most inguinal hernias contain omental fat or small bowel loops, rare variants, such as the vermiform appendix, may harbor unexpected contents. Amyand's hernia, first described by Claudius Amyand in 1735, represents a unique subset of inguinal hernias wherein the appendix is found within the hernia sac [2]. The reported incidence of Amyand's hernia is exceedingly low, estimated to occur in less than 1% of all inguinal hernias [3]. Despite its rarity, Amyand's hernia presents diagnostic and therapeutic challenges for surgeons due to its variable clinical presentation and management implications. While some cases may remain asymptomatic, others can manifest as acute appendicitis within the hernia sac, leading to complications such as perforation and abscess formation [4].

The diagnosis of Amyand's hernia is often incidental, made intraoperatively during hernia repair or preoperatively with imaging studies such as ultrasonography or computed tomography. Management strategies for Amyand's hernia depend on several factors, including appendiceal inflammation, the patient's clinical condition, and the surgeon's expertise [5]. In cases where the appendix is found incidentally without signs of inflammation, the hernia contents and hernia repair can be reduced without appendectomy. However, in cases of acute appendicitis within the hernia sac or suspicion of appendiceal pathology, appendicectomy is indicated along with hernia repair [6]. Surgical techniques for repairing Amyand's hernia vary and may include open or laparoscopic approaches, with or without mesh reinforcement [7]. The choice of surgical technique depends on factors such as the surgeon's preference, the patient's comorbidities, and the complexity of the hernia presentation. Despite advancements in surgical techniques, managing Amyand's hernia remains a topic of debate, and optimal management strategies continue to evolve [8].

## Case presentation

A 45-year-old man presented to our outpatient department with complaints of right-sided inguinoscrotal swelling that had been progressively increasing over the past year. The patient reported that the swelling became more prominent during activities such as straining and coughing, which tended to reduce when he lay down. On physical examination, a reducible mass was palpated in the right inguinal region, consistent with an indirect inguinal hernia. There were no signs of inflammation or tenderness over the swelling, and the patient did not report any associated symptoms such as pain or bowel disturbances.

Routine blood investigations were within normal limits, including complete blood count and inflammatory markers. Ultrasonography of the inguinoscrotal region was performed, which revealed a right inguinal hernia with a defect measuring approximately 1 cm. The hernia sac was found to contain omentum as content without any evidence of incarcerated or strangulated bowel loops. Based on the clinical and radiological findings, a diagnosis of a right indirect inguinal hernia was made.

**Figure 1:**
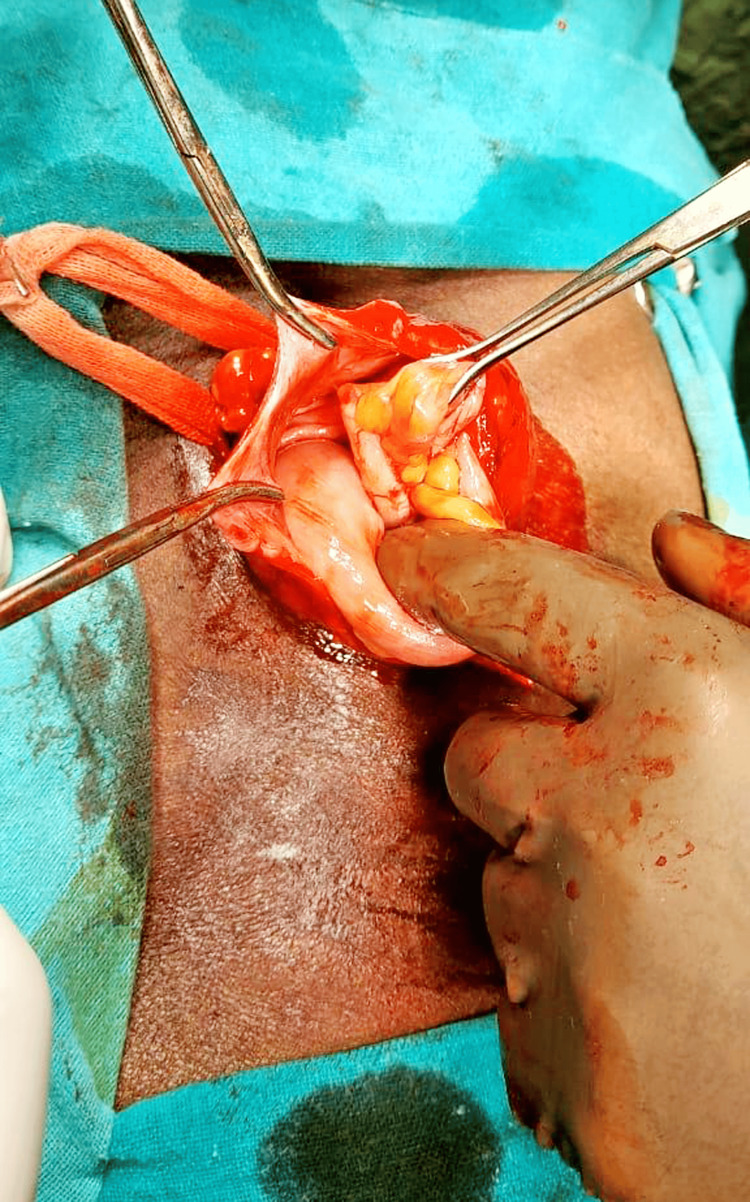
Hernia sac at the time of operation

The patient was counseled regarding the diagnosis and the need for surgical intervention to repair the hernia. After obtaining informed consent, he was scheduled for elective right-sided Lichtenstein repair. In the operating room, under spinal anesthesia, a standard incision was made over the right inguinal region, and dissection of the inguinal canal was performed. Upon identification of the hernia sac, it was carefully dissected and opened (Figure 1).

**Figure 2:**
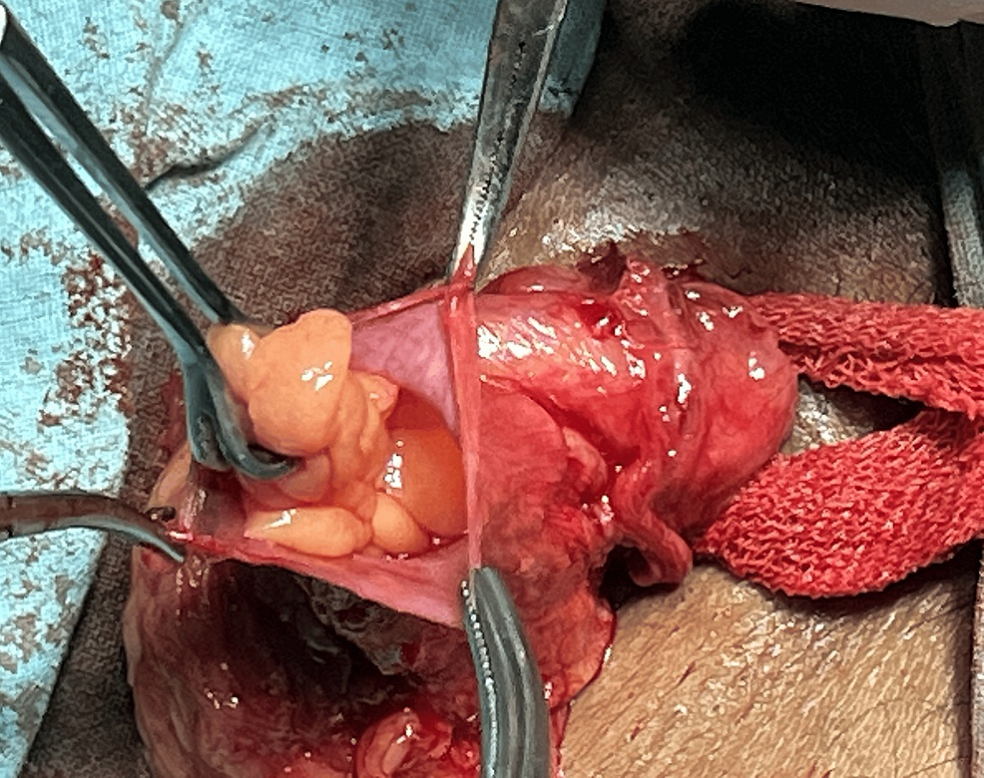
The sac contained both the omentum and the vermiform appendix

To our surprise, intraoperatively, the sac contained both the omentum and the vermiform appendix (Figure 2). However, the appendix did not show any signs of inflammation or necrosis. Hernia contents were carefully reduced, and the appendix was gently returned to the abdominal cavity. The Liechtenstein meshplasty procedure was then completed without complications, and the mesh was secured over the inguinal canal to reinforce the weakened area.

Postoperatively, the patient had an uneventful recovery period and was closely monitored for any signs of surgical site infection or recurrence of hernia. He was discharged home with appropriate postoperative instructions and scheduled for follow-up visits in the outpatient clinic. At the follow-up appointments, the patient remained asymptomatic with no evidence of hernia recurrence or complications related to the surgery. He expressed satisfaction with the procedure's outcome and resumed his usual daily activities without any limitations.

## Discussion

Amyand’s hernia, a rare inguinal hernia characterized by the presence of the vermiform appendix within the hernia sac, represents a unique diagnostic and management challenge for surgeons. Although inguinal hernias are common surgical presentations, the incidence of Amyand’s hernia is exceptionally low, estimated to occur in less than 1% of all cases of inguinal hernias [9]. This rarity underscores the importance of considering this differential diagnosis, especially in cases of atypical inguinal hernias.

In our case, the diagnosis of Amyand’s hernia was made incidentally during surgical exploration for inguinal hernia repair. The intraoperative finding of the omentum and the vermiform appendix within the hernia sac was unexpected but consistent with the literature, where the contents of Amyand’s hernia can vary. It may include a normal appendix, an inflamed appendix, or other abdominal viscera [10]. Despite the presence of the appendix within the hernia sac, there were no signs of inflammation or necrosis, allowing for a conservative surgical approach without the need for appendectomy.

The management of Amyand’s hernia depends on various factors, including appendiceal inflammation, the patient’s clinical condition, and the surgeon’s experience. In cases of incidental finding without appendicitis, as in our case, the hernia contents and hernia repair can be reduced without appendectomy [11]. However, in cases of acute appendicitis within the hernia sac or suspicion of appendiceal pathology, appendectomy is indicated to prevent potential complications such as perforation and peritonitis [11].

The choice of surgical technique for inguinal hernia repair in the setting of Amyand’s hernia is also crucial. In our case, we opted for the Liechtenstein meshplasty procedure, which is a well-established technique for inguinal hernia repair with low recurrence rates and favorable outcomes [12]. Using mesh reinforcement helps strengthen the weakened inguinal canal and reduce the risk of hernia recurrence, particularly in cases where the hernia sac contains non-intestinal contents such as the appendix.

## Conclusions

In conclusion, Amyand’s hernia remains an uncommon yet intriguing clinical entity that challenges surgeons with its varied presentations and management dilemmas. Our case underscores the importance of maintaining a high index of suspicion for this condition, especially when encountering atypical inguinal hernias. The successful outcome of our surgical intervention without appendectomy highlights the significance of individualized treatment approaches based on intraoperative findings and patient characteristics. Continued reporting of cases and further research efforts are warranted to refine diagnostic criteria, optimize surgical strategies, and improve outcomes for patients with Amyand’s hernia. Through enhanced understanding and tailored management, clinicians can strive to achieve optimal outcomes and minimize complications in this rare but clinically significant condition.
